# Changing practice of macular hole surgery in the UK (2017–2024)

**DOI:** 10.1038/s41433-025-04238-7

**Published:** 2026-02-17

**Authors:** Xiaoman S. Li, David Yorston, David H. Steel

**Affiliations:** 1 Biosciences Institute, Newcastle University, Newcastle upon Tyne, UK; 2 Tennent Institute of Ophthalmology, Gartnavel Hospital, Glasgow, UK; 3 Sunderland Eye Infirmary, South Tyneside and Sunderland NHS Foundation Trust, Sunderland, UK; 4 Hull University Teaching Hospitals NHS Trust, Kingston upon Hull, UK; 5 Middle and South Essex NHS Foundation Trust, Basildon, UK; 6 Lancashire Teaching Hospitals NHS Trust, Preston, UK; 7 University Hospital Dorset NHS Foundation Trust, Poole, UK; 8 University Hospital Sussex NHS Foundation Trust, Brighton, UK; 9 Manchester University NHS Foundation Trust, Manchester, UK; 10 Liverpool University Hospitals NHS Foundation Trust, Liverpool, UK; 11 James Paget University Hospital NHS Foundation Trust, Great Yarmouth, UK; 12 Guy’s and St Thomas’ NHS Foundation Trust, London, UK; 13 East Suffolk and North E NHS Trust, Colchester, UK; 14 Oftalmologico Recolestas-IOR, Castilla y León, Spain; 15 Brighton and Sussex University Hospitals NHS Trust, Brighton, UK; 16 Somerset NHS Foundation Trust, Taunton, UK; 17 Maidstone and Tunbridge Wells NHS Trust, Tunbridge Wells, UK; 18 Hywel Dda University Health Board, Carmarthen, UK; 19 University Hospitals of North Midlands NHS Trust, Stoke-on-Trent, UK; 20 York and Scarborough Teaching Hospitals NHS Foundation Trust, Scarborough, UK; 21 Taunton and Somerset NHS Foundation Trust, Taunton, UK; 22 Hospital Universitario Ramón y Cajal, Madrid, Spain; 23 The Royal Wolverhampton NHS Trust, Wolverhampton, UK; 24 Sheffield Teaching Hospitals NHS Foundation Trust, Sheffield, UK; 25 Newcastle Hospitals NHS Foundation Trust, Newcastle, UK; 26 Ninewells Hospital, NHS Tayside, Dundee, UK; 27 The Forbury clinic, Reading, UK

**Keywords:** Retinal diseases, Health services

## Introduction

Idiopathic full-thickness macular holes (iFTMHs) are a common cause of visual impairment in the UK. Recent Royal College of Ophthalmologists (RCOphth) guidelines highlighted the importance of timely surgery and the benefits of internal limiting membrane (ILM) flaps in larger holes [1, 2]. To provide a baseline for future comparisons and to assess recent change pre and post COVID-pandemic, we carried out an audit using nationally collected data.

## Methods

We reviewed 2,152 consecutive iFTMH surgeries recorded in the BEAVRS/EURETINA database across four one-year periods (2017, 2019, 2021, 2024). Data were prospectively entered immediately after surgery and at ≥ 2-month follow-up. Statistical tests included ANOVA or Kruskal-Wallis tests for continuous variables (depending on distribution), Welch’s *t*-test for comparison between two independent groups, and the Chi-Square test for categorical data.

## Results

### Presenting features

Baseline characteristics were stable across years (mean age 70, 68–73% female). Surgeon numbers and case volumes per surgeon were consistent. Mean minimum linear diameter (MLD) increased from 395 µm in 2017 to 420 µm in 2021 (*p* = 0.049), and the proportion of large holes (>500 µm) rose from 26% to 31% (*p* = 0.003), remaining high in 2024 (Table 1). Symptom duration lengthened post-pandemic (6.3 to 10.1 months, 2017 versus 2021) but improved to 4.6 months by 2024 (*p* < 0.001). However, the interval between assessment and operation doubled between 2017 and 2024 (1.2 vs 2.4 months, *p* < 0.001). Presenting visual acuity (VA) remained stable.

**Table 1:** Summary of clinical data.

	2017	2019	2021	2024	*P*
Total number (*n*)	510	644	608	553	--
Number of surgeons per year (*n*)	29	34	32	28	--
Cases per surgeon, Median (IQR)	16.0 (8–23)	16.5 (9–24)	15.0 (8–28)	14.5 (8–20)	0.721
**Baseline features**					
Age, years. Mean (SD)	70.1 (6.7)	69.9 (6.5)	70.8 (6.7)	69.6 (8.2)	0.084
Sex, female *n* (%)	347 (68.0%)	444 (68.9%)	412 (67.8%)	387 (70.0%)	0.851
Baseline Va, logMAR Median (IQR)	0.8 (0.8–1)	0.8 (0.7–1)	0.8 (0.7–1)	0.8 (0.6–1)	0.834
MLD, microns Mean (SD)	395 (149)	399 (162)	420 (160)	407 (186)	0.043^*^
MLD < 500, *n* (%)	390 (76.5%)	476 (73.9%)	417 (68.6%)	386 (69.8%)	0.011^*^
MLD ≥ 500, *n* (%)	120 (23.5%)	168 (26.1%)	191 (31.4%)	167 (30.2%)	0.011^*^
Symptom duration, months Mean (SD)	6.3 (4.4)	7.3 (8.1)	10.1 (30.2)	4.6 (5.7)	<0.001^*^
Symptom duration > 6 months, *n* (%)	101/251 (40.2%)	155/297 (52.2%)	150/255 (58.8%)	133/553 (24.1%)	<0.001^*^
Symptom duration > 12 months, *n* (%)	22/251 (8.8%)	33/297 (11.1%)	50/255 (19.6%)	48/553 (8.7%)	<0.001^*^
Time between assessment and operation, months Mean (SD)	1.2 (1.4)	2.0 (1.8)	2.3 (2.4)	2.4 (2.3)	<0.001^*^
**Surgery**					
ILM peel, complete *n* (%)	485 (98.8%)	596 (96.0%)	578 (96.7%)	522 (94.4%)	0.002^*^
ILM flap, *n* (%)	9 (1.8%)	29 (4.5%)	79 (13.0%)	149 (26.9%)	<0.001^*^
ILM flap in MLD > 500, *n* (%)	3 (2.7%)	19 (11.4%)	64 (34.0%)	99 (60.7%)	<0.001^*^
**Outcomes**					
Anatomically closed, *n* (%)	497/510 (97.5%)	626/644 (97.2%)	577/608 (94.9%)	476/495 (96.2%)	0.076
< 500 microns, *n* (%)	387/390 (99.2%)	471/476 (98.9%)	401/417 (96.2%)	346/351 (98.6%)	0.003^*^
≥ 500 microns, *n* (%)	110/120 (91.7%)	155/168 (92.3%)	176/191 (92.1%)	130/144 (90.3%)	0.919
Anatomically closed, > 500 microns	--				0.036^*^
ILM flap, *n* (%)		149/155 (96.1%)			
No ILM flap, *n* (%)		326/362 (90.1%)			
Postop Va, logMAR Median (IQR)	0.4 (0.2–0.6)	0.4 (0.3–0.6)	0.5 (0.3–0.6)	0.5 (0.3–0.6)	<0.001^*^

^*^Statistically significant difference (*p* < 0.05)

### Surgical approach

Rates of complete ILM peel were consistently high (>95%), whereas ILM flap use rose sharply: from 2% in 2017 to 27% in 2024 (*p* < 0.001). Among large holes, ILM flap adoption rose from 3% in 2017 to 61% in 2024 (Chi-square trend = 8.6, *p* = 0.010).

### Outcomes

Overall anatomical closure rates remained high across all years (>95%). Among large holes, closure improved significantly with ILM flap use compared to those without (96% vs 90%, *p* = 0.032). Median follow-up VA worsened post-pandemic (0.4 vs 0.5 logMAR, 2019 vs 2024, *p* < 0.001).

## Discussion

Significant changes in iFTMH management were observed immediately post-pandemic, which have not fully recovered. Longer symptom duration and surgical waiting times between 2019 and 2021 likely reflected COVID-related delays, contributing to larger macular hole size and static anatomical outcomes despite wider adoption of ILM flaps [1, 3]. Presenting VA remained stable, but poorer follow-up VA persisted from 2021 onwards.

Subsequent improvements in symptom duration by 2024 may reflect earlier referral through mitigating strategies [4]. However, surgical waiting time remains prolonged and the proportion of large holes is high. Anatomical closure rates and visual outcomes have not yet returned to pre-pandemic levels, possibly due to persistent delays or a lag between practice change and measurable clinical impact.

In summary, iFTMHs presented later and larger in the immediate post-COVID period. Despite improvements in referral pathways, surgical waiting times and the proportion of larger holes remain elevated. This contributes to an ongoing plateau in anatomical outcomes and decline in follow-up VA, despite wider ILM flap use. We highlight the continued importance of timely surgery and analysis of emerging data to assess whether trends will begin to normalise with practice change.

## Data Availability

XSL, DY, and DHS have had full access to all the data in the study and take responsibility for the integrity of the data. The data sets generated and/or analysed in the current study are not publicly available.

## References

[1] Idiopathic Full Thickness Macular Holes [Internet]. The Royal College of Ophthalmologists. [cited 2025 Oct 8]. Available from: https://www.rcophth.ac.uk/resources-listing/idiopathic-full-thickness-macular-holes/

[2] Tzoumas N, McNally TW, Teh BL, Zaman M, Yorston D, Lois N, et al. Internal limiting membrane flaps in macular hole surgery: a systematic review and individual participant data meta-analysis. Ophthalmol Retina. 2025;9:717–30. 39923898 10.1016/j.oret.2025.02.003

[3] Murphy DC, Al-Zubaidy M, Lois N, Scott N, Steel DHMacular Hole Duration Study Group. The effect of macular hole duration on surgical outcomes: an individual participant data study of randomized controlled trials. Ophthalmology. 2023;130:152–63. 10.1016/j.ophtha.2022.08.028 36058348

[4] Raimondi R, Ghazal D, Steel DH. Reducing time to surgery in patients affected by idiopathic full thickness macular holes. Eye. 2023;37:3509–10. 37019995 10.1038/s41433-023-02517-9 PMC10630444

